# Sensing with Molecularly Imprinted Membranes on Two-Dimensional Solid-Supported Substrates

**DOI:** 10.3390/s24165119

**Published:** 2024-08-07

**Authors:** Lishuang Wang, Nan Li, Xiaoyan Zhang, Ivan Bobrinetskiy, Ivana Gadjanski, Wangyang Fu

**Affiliations:** 1 School of Pharmaceutical Sciences, Capital Medical University, Beijing 100069, China; lishuangwang@mail.ccmu.edu.cn (L.W.); ln0828@mail.ccmu.edu.cn (N.L.); 2 BioSense Institute, University of Novi Sad, Dr Zorana Đinđića 1a, 21000 Novi Sad, Serbia; bobrinet@biosense.rs (I.B.); igadjanski@biosense.rs (I.G.); 3 School of Materials Science and Engineering, Tsinghua University, No. 1 Tsinghua Yuan, Haidian District, Beijing 100084, China

**Keywords:** molecularly imprinted membranes, biochemical sensors, all-solid-state, ion-sensitive field-effect transistor, point-of-care testing

## Abstract

Molecularly imprinted membranes (MIMs) have been a focal research interest since 1990, representing a breakthrough in the integration of target molecules into membrane structures for cutting-edge sensing applications. This paper traces the developmental history of MIMs, elucidating the diverse methodologies employed in their preparation and characterization on two-dimensional solid-supported substrates. We then explore the principles and diverse applications of MIMs, particularly in the context of emerging technologies encompassing electrochemistry, surface-enhanced Raman scattering (SERS), surface plasmon resonance (SPR), and the quartz crystal microbalance (QCM). Furthermore, we shed light on the unique features of ion-sensitive field-effect transistor (ISFET) biosensors that rely on MIMs, with the notable advancements and challenges of point-of-care biochemical sensors highlighted. By providing a comprehensive overview of the latest innovations and future trajectories, this paper aims to inspire further exploration and progress in the field of MIM-driven sensing technologies.

## 1. Introduction

Molecular-specific recognition plays a crucial role in regulating biological systems, involving organic functional groups characterized by distinct chemical or biological properties. These groups interact via highly specialized and efficient bonding methods, resulting in unique biological effects manifested as physiological activities or physicochemical interactions with the external environment [1,2,3,4,5]. Over time, natural systems have evolved an array of specific affinities to meet functional demands, exemplified by interactions such as enzyme–inhibitor complexes [6,7], sugar–lectins [8], RNA–ribosomes [9,10,11], and antibody–antigen pairings [12,13]. Precise molecular recognition is essential for biological processes and represents a sophisticated form of sensing. Given its importance in catalysis, separation, and detection, this area has become a central focus of chemical research. Although a biosystem is capable of producing antibodies for a wide range of foreign entities, employing receptors in chemical processes leads to difficulties such as high expenses and vulnerability to environmental factors [14]. Replicating the specific ligand recognition found in biological systems presents a significant challenge for researchers. Inspired by natural receptor–ligand interactions, scientists have developed molecular imprinting technology (MIT). This technique involves the formation of synthetic polymers with highly specific cavities designed for target molecules, thereby mimicking natural recognition systems [15,16,17].

Wulff and Klotz pioneered the concept of imprinting with organic polymers, coining the term “molecularly imprinted polymers” (MIPs) in 1972. The initial MIPs were conceptualized using a silica-based imprinting approach [18]. Over the decades, spherical MIPs have evolved and gained popularity due to their ability to selectively recognize target molecules within complex mixtures. Researchers have utilized spherical silica [19], quantum dots [20], and magnetic particle nanoparticles [21] as substrates to prepare molecularly imprinted particles with core-shell structures. However, spherical MIPs have limitations, including relatively low binding properties and restricted binding signaling mechanisms. Consequently, a specialized membrane that recognizes specific molecules, termed a molecularly imprinted membrane (MIM), was developed [22,23]. MIMs exemplify a unique variety of imprinted polymers that merge particular recognition attributes with conventional aspects of membrane technology.

Furthermore, MIMs can be combined with various two-dimensional (2D) solid-supported substrates to enhance their performance and broaden the application field of MIT [24,25,26]. The huge specific surface area and high porosity of 2D solid-supported substrates provide more recognition sites, enabling the better separation and detection of target molecules. Additionally, 2D solid-supported substrates serve as excellent support structures, simplifying the preparation process of MIMs and allowing precise control over the membrane thickness, pore structure, and surface chemistry, thus modulating membrane properties. Numerous reports have detailed the combination of MIMs with 2D solid-supported substrates to enable coupling with electrical, optical, and mechanical detection techniques [27,28,29].

**Figure 1:**
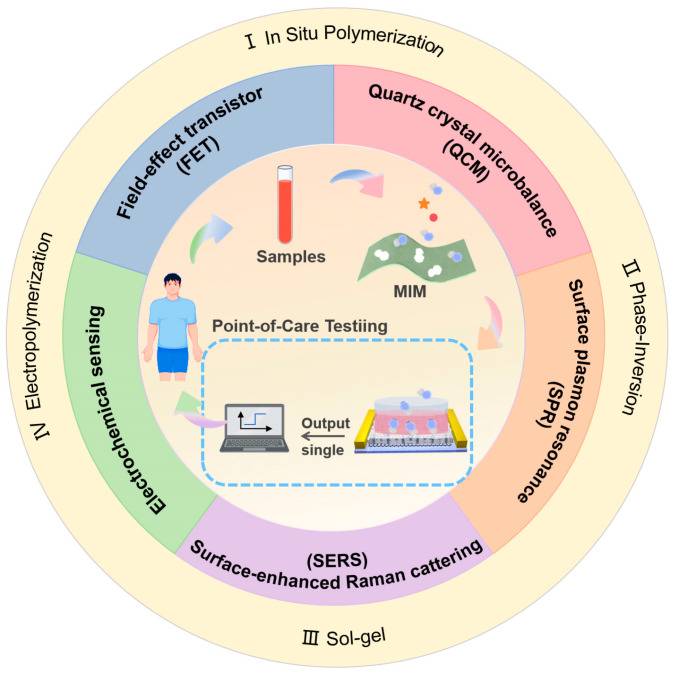
Overview of the principle and classification for molecularly imprinted membranes (MIMs) based on 2D solid-supported substrates for point-of-care testing via integration with electrochemical sensing, FET, QCM, SPR, and SERS technologies.

Compared to naturally derived ligands like antibodies, MITs have become prominent in materials science focused on molecular affinity. MITs offer cost-effectiveness, adaptability, and superior stability. This review progresses from discussing MIT particles in solution—including their state-of-the-art advancements, benefits, and limitations—to exploring the development of MIT membranes on solid surfaces. The aim is to introduce the applications of solid-supported MIMs in various fields, with a particular focus on sensing. We will primarily discuss and summarize the latest technological advancements and applications of MIMs in combination with electrochemistry sensing, surface-enhanced Raman scattering (SERS), surface plasmon resonance (SPR), and quartz crystal microbalance (QCM) technologies. Additionally, we will explore future prospects for innovative applications of MIMs in field-effect transistor biochemical sensors. Figure 1 shows the basic principle and classification of MIMs based on 2D solid-supported substrates.

### 1.1. Molecular Imprinting Technology

MIT involves the polymerization and assembly of specifically designed monomers around a target molecule, creating a customized “artificial lock” that can specifically recognize the “key” (template). This technology has its origins in 1931, when Polyakov first reported the binding of silica particles derived from sodium silicate to target molecules in the presence of organic solvents like benzene, toluene, or xylene. Theoretically, MIPs can be customized to target a diverse array of target analytes, such as ions, proteins, and peptides [5,30]. For instance, a recent study introduced a novel method that is enzyme-free, cost-effective, and user-friendly for detecting α-fetoprotein (AFP) and carcinoembryonic antigen (CEA) cancer biomarkers built on fluorescent molecularly imprinted coupled polythiophene (FMICP) [31]. The sensors exhibited a high sensitivity to CEA concentrations within the range of 0.001 to 200 ng/mL, with detection limits of 40 fg/mL and 3.5 fg/mL, respectively. Additionally, a distinctive double-imprinted electrochemical paper-based analytical instrument (Di-ePAD) was developed, capable of concurrently detecting 8-hydroxy-20-deoxyguanosine (8-OHdG) and 3-nitrotyrosine (3-NT) in a complex matrix (urine and plasma), along with oxidation and nitrification biomarkers [32].

### 1.2. Development of Molecular Imprinting Membranes

Piletsky et al. initially employed MIMs to selectively separate adenosine monophosphate (AMP) and guanosine monophosphate (GMP) in 1990 [33]. Subsequently, Mathew-Klotz et al. independently prepared MIMs using 9-ethyladenine, which served as a template, and AIBN, which acted as an initiator, by polymerizing glycol dimethacrylate and methyl methacrylate. They further selected adenosine for the infiltration of adenosine from an adenosine–guanosine mixture [34]. Following this, a novel type of imprinted membrane for theophylline (THO) using the phase-inversion method was developed [35]. Polyacrylonitrile–acrylic acid copolymers were prepared using THO molecules as template molecules and dimethylsulfoxide (DMSO) copolymers as functional monomers by the phase-inversion precipitation method. The selectivity was confirmed through the structural analogue of caffeine (CAF), highlighting the specific hydrogen bonding between the THO molecule and the polyacrylonitrile–co-acrylic acid group.

The synthesis process of MIMs closely mirrors that of MIPs. Covalent or non-covalent template molecules are introduced during the synthesis process of a membrane, followed by the leaching out of the template, leaving specific pores in the membrane with an affinity for template-like molecules [23]. Initially, MIMs could only selectively identify target molecules in solution through simple static adsorption or osmosis via membrane devices [23]. However, with advancements in science and technology, MIMs can now imprint a variety of template analytes, such as inorganic ions, drugs, nucleic acids, proteins, viruses, and even cells [36,37,38,39,40]. Numerous reports have detailed the combination of MIMs with 2D solid-supported substrates to enable coupling with electrical, optical, and mechanical detection techniques. For instance, the combination of MIMs with semiconducting substrates facilitates the development of ultrasensitive FET biochemical sensors, the promising application of which will be discussed in the prospect part of this review. Although electrochemical sensors may not match FET sensors’ sensitivity, they have become popular in recent years due to their simplicity, ease of production, and low equipment costs. Furthermore, electrochemical sensors necessitate less complex electrode preparation than FET sensors [41,42]. Electrodes can also be coated with a single layer of MIMs. There are numerous techniques for covering an electrode’s surface with MIMs, including electropolymerization. The sensing signal depends on the rate of mass transfer of the electrochemically active analyte to the MIM-covered electrodes. In addition to electrical coupling, MIMs can also be combined with optics, mechanics, and other disciplines. Optical MIPs sensors, often designated as molecularly imprinted photosensitive materials (MIOMs), can be applied to the sensing of both gas and liquid phases [43,44,45]. These optical sensors typically detect the specific fluorescence signal of the object under measurement. Furthermore, QCM technology, as a piezoelectric mass sensing method, is simple to operate and enables the accurate detection of resonant frequency changes associated with mass loading [46,47]. By merging molecular imprinting with a slender piezoelectric quartz crystal, which is layered between two metallic contacts, the attachment of the desired molecule to the surface of the MIMs leads to an increase in the crystal’s mass, consequently altering its resonant frequency.

## 2. Preparation Methods for MIMs

There are two primary types of chemical interactions that play a role during the development process of polymers with molecular imprinting techniques: covalent bonding and noncovalent associations [48,49]. Covalent interactions, which were introduced by Wulff, rely on covalent bonds that can be reversed between template and functional monomers within pre-polymerized blends [18]. Following polymerization, these bonds are chemically broken to release and remove the template. In the recombination process, covalent bonds are formed through interactions involving functional monomers and the template. Covalent imprinting involves the formation of reversible covalent bonds between functional monomers and target analytes. Examples include carboxylate bonds, borate bonds [50], and Schiff bases [51]. In contrast, non-covalent molecular imprinting, as introduced by Mosbach and Sellergren, utilizes non-covalent interactions of templates and monomers. These engagements encompass hydrogen bonding, hydrophobic interactions, van der Waals forces, and metal chelation [52,53,54,55]. During polymerization, a solvent–template–monomer–cross-linker–initiator system is used to ensure the precise spatial arrangement of these components. Afterward, tailored solvents are employed to remove the target molecules, resulting in the creation of the target three-dimensional MIPs.

**Figure 2:**
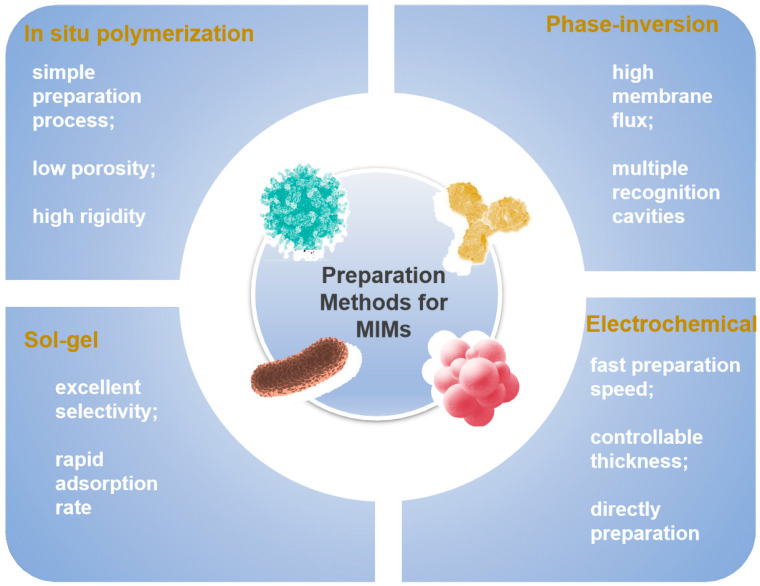
The conceptual overview of the main synthetic methods covered in this review, which includes the merits of each method.

Each of these synthesis types has its own advantages and disadvantages. The covalent method allows for control over the stoichiometry of imprinted materials, resulting in more uniform identification sites compared to the non-covalent method. Covalently imprinted polymers are known for their stability and selectivity. However, they have limitations such as a restricted number of functional groups interacting with the target molecule and slow binding dynamics. Additionally, their reuse in cleaving and rebinding templates can be challenging due to these limited interactions. Furthermore, covalent interactions differ from the natural recognition mechanisms observed in biological systems at the molecular level. They replicate the size, shape, and chemical groups of template molecules leading to a high specificity in molecular detection. On the other hand, non-covalent methods rely on weaker template–functional monomer interactions, which are not as robust as covalent bonds. This allows for a broader range of imprinting targets. This method benefits from the ease with which monomer/template complexes can form through self-assembly, with the template being released under gentle conditions, potentially yielding a high density of recognition cavities [56]. Currently, non-covalent MIT is the most commonly employed due to its simple synthesis process, applicability to various templates, and compatibility with many functional monomers. The synthesis methods of MIMs are continually expanding based on non-covalent binding methods. Each synthesis method possesses distinct characteristics. Figure 2 contains the current most common synthesis methods and summarizes their advantages. For instance, the in situ polymerization method offers a straightforward preparation process, the phase-inversion method enhances membrane flux, the sol-gel method demonstrates excellent selectivity, and the electrochemical method boasts a rapid preparation speed. The comprehensive examination of the advantages and disadvantages associated with each method of preparation, along with its corresponding procedure, can be found in Table 1.

### 2.1. In Situ Polymerization Method

**Figure 3:**
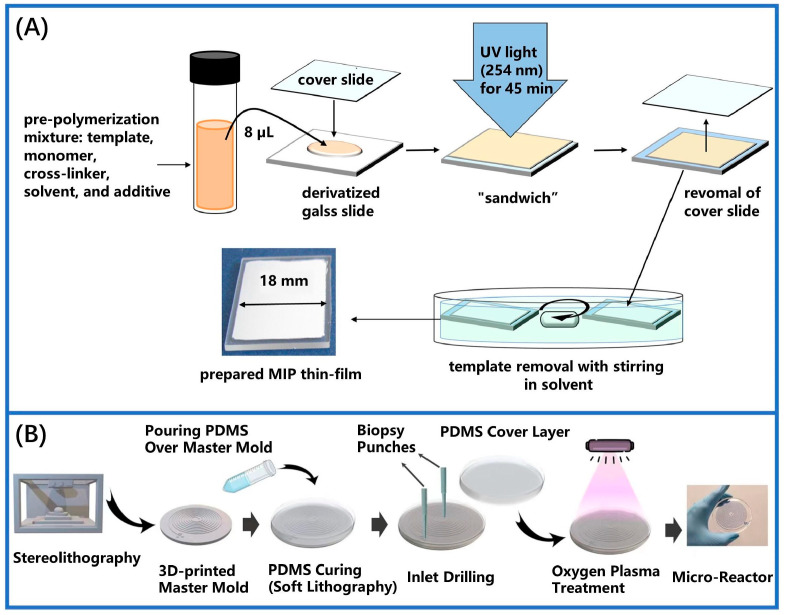
The synthetic process of MIMs by in situ polymerization. (**A**) The MIM was synthesized using photo-radical polymerization on a thin solution layer composed of liquid pre-polymerization solution, which was sandwiched between a derivatized and a quartz cover glass slide [73]. (**B**) In situ synthesis of MIM in a microreactor [58].

Piletsky et al. pioneered the successful development of MIMs, selecting adenylate as a template molecule through in situ polymerization in 1990 [33]. This method involves dissolving and mixing a precise ratio of template molecules, functional group monomers, and cross-linkers between two plates. The cross-linking reaction is initiated by UV irradiation or heat, which promotes direct polymerization on the carrier surface, resulting in the formation of an MIM [57,71,72]. As illustrated in Figure 3A, a custom porous MIM was created using in situ photo-radical polymerization conducted on a slide. This technique offers a viable method for selectively detecting trace polycyclic aromatic sulfur heterocycles (PASHs) in seawater [73]. Subsequently, researchers prepared additional MIMs using in situ photo-radical polymerization on a glass plate [58]. These membranes were utilized as microextraction adsorbents to detect trace levels of polycyclic aromatic sulfur heterocycles of seawater, achieving a detection limit between 0.029 and 0.166 μg/L. As illustrated in Figure 3B, MIPs were synthesized through in situ polymerization within a small reaction vessel. This approach accelerates reaction rates and allows for controlled in situ growth, with the continuous synthesis of trillions of MIP nanoparticles taking approximately 5 to 30 min [58]. Additionally, a straightforward, rapid, and highly sensitive method was devised for determining artemisinin (ART) using electrochemical sensors [74]. The optimization of the MIM on the graphene sensor was achieved by using acrylamide (AM) as the functional monomers. The wrinkled structure of the graphene enhanced the sensor’s construction by providing a large surface area for the MIM to serve as a recognition area. Concurrently, the research group expanded their investigation into the structure of a Pd electrochemical sensor [75]. The polymerization process was completed by applying the solution to the electrode surface, covering it with glass, and using an infrared lamp for heating. Although this preparation method is simple and manageable, the extensive degree of cross-linking results in high membrane rigidity and minimal porosity. This, in turn, leads to reduced membrane flux and increased mass transfer resistance, thereby limiting the practical application of membranes prepared via in situ polymerization.

### 2.2. Phase-Inversion Method

**Figure 4:**
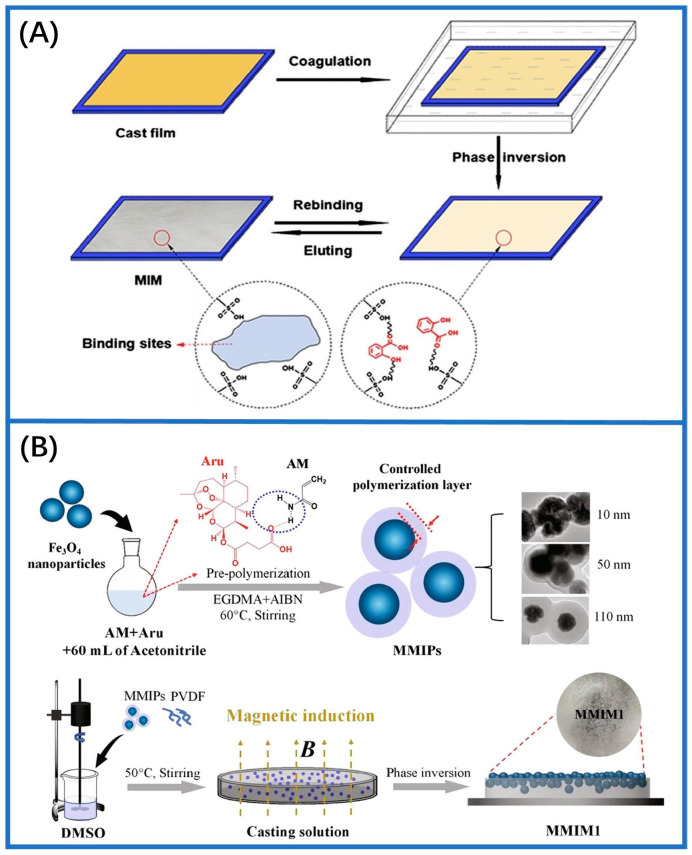
The technique of phase inversion employed in the fabrication of MIMs. (**A**) Schematic representation of a hybrid MIM made from cellulose acetate (CA) prepared by phase inversion for detecting salicylic acid (SA) [78]. (**B**) Manufacturing MIMs through the phase-inversion technique, facilitated by magnetic field forces [62].

Phase-inversion technology entails dissolving templates, membrane materials, cross-linkers, and functional monomers mixed in an appropriate solvent. The resulting solution is then applied onto a carrier, such as clean glasses. Bidirectional diffusion occurs between the casting and precipitating solution, leading to the phase separation of the casting solution from the uniform mixture and complete detachment. This process results in MIMs with specific molecular recognition capabilities. Currently, phase-inversion techniques include dry phase, thermogelation, wet phase, and reverse phase using water vapor inhalation [60,61]. Recently, researchers prepared MIMs by combining polystyrene with polypeptide recognition groups containing matrix polymers via the “dry reverse transfer” method. The resulting membranes demonstrated a higher permeability compared to that of non-imprinted membranes (NIMs) [76]. Early studies utilizing phase-inversion technology for MIMs employed cellulose diacetate as a matrix polymer. Optimization efforts concentrated on the solvent, reagent ratio, and total polymer concentration to improve membrane effectiveness and achieve molecular imprinting effects [77]. Figure 4A shows the MIMs for salicylic acid (SA) created by the reverse transfer technique. After first preparing the casting solution containing the template molecules, the solution was cast on a polished glass plate at room temperature. The glass plate was swiftly submerged in a gelling bath after 30 s. The phase transition between the polymer and deionized water then commences at 18 °C [78]. As depicted in Figure 4B, researchers developed an innovative MIM by the phase-inversion technique [62]. In subsequent research, a highly efficient ion-imprinted membrane was created employing the non-solvent-induced phase separation (NIPS) technology with ionized platinum (IV) serving as the template. This approach achieved the selective separation of platinum (IV) [79]. Additionally, poly(vinylidene fluoride) (PVDF) composite membranes were produced using the inverse transference method, which demonstrated a strong rebinding capability (31.25 mg/g) in adsorption tests with environmental effluents [80]. The primary distinction between phase transition and in situ polymerized methods for membrane fabrication lies in the incorporation of recognition sites into the polymer. In phase transition, the recognition sites are directly introduced into the polymer. MIMs are created from the polymer solution with target molecules present. Throughout the imprinting process, both recognition binding sites and the penetration channels are formed. The phase-inversion process has been demonstrated to be partially influenced by kinetic and thermodynamic factors and typically is completed within a few milliseconds. Therefore, understanding the mechanism of this process poses challenges due to its complexity.

### 2.3. Sol-Gel Method

**Figure 5:**
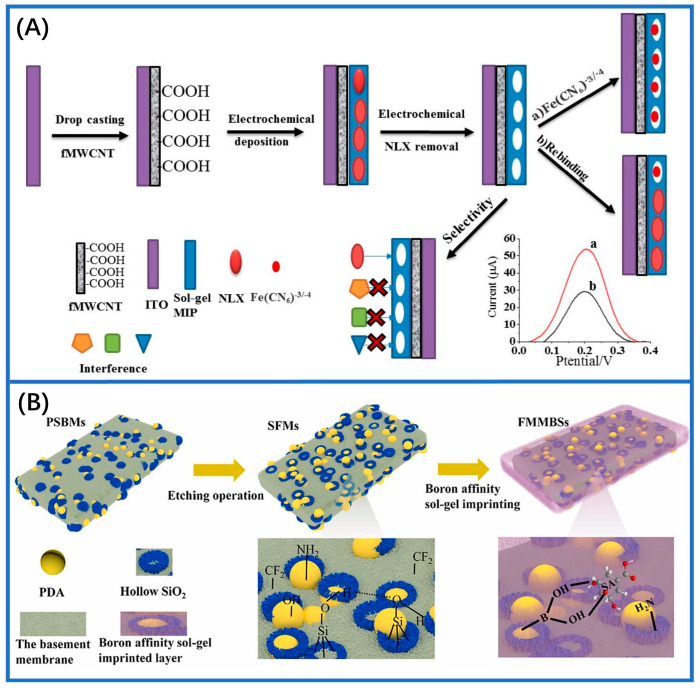
Preparation of MIMs by sol−gel polymerization. (**A**) The schematic illustrates the fabrication of an MIP sensor and the naloxone assay using the sol−gel method [81]. (**B**) Preparation process for preparing boron−affinity sol−gel MIM [82].

The sol-gel method for preparing MIMs involves constructing films by incorporating templates into network structures composed of inorganic and organic inorganic. This technique has seen significant advancement in recent years and is increasingly utilized in mixture separation and sensor development. Generally, functional group monomers and cross-linking agents are initially dissolved together, followed by the addition of template compounds. Subsequently, a polymer network forms through polymerization or cross-linking reactions. Once the template molecules are removed, MIMs are formed. This technology involves a gentle reaction environment and is straightforward to execute. Additionally, during the reaction process, the properties of the synthesized film can be finely tuned through precisely controlling the reagent composition, density, and pH value belonging to the reaction solution [63,64,65]. Figure 5A depicts the MIP-modified electrode prepared using the sol-gel technique for the electrochemical detection of the drug naloxone (NLX). In this setup, pyrrole imparts conductivity to the sensor, while triethoxyphenylsilane (TEPS) functions as the functional group monomer and tetraethoxysilane (TES) acts as the cross-linker. This sensor is excellent for the quantitative testing of NLX in artificial urine samples [81]. Figure 5B illustrates the process of preparing borate affinity sol-gel molecular imprinting. This approach enhances selectivity and binding affinity through the synergistic interaction of imprinted cavities and boric acid, achieving an imprinting factor greater than 10 [82]. Additionally, another study focused on developing sensing membranes by adding microcystin-LR templates into the sol-gel matrix [64]. During the hydrolysis of the mixture, the substrate undergoes polymerization, forming a network. Ultimately, the templates were extracted from the imprinted polymer, creating selectively imprinted cavities behind it. In another investigation, molecularly imprinted sol-gel hollow-fiber membranes (MSHMs) was used to detect horse uric acid from plasma and urine samples by the sol-gel method [66]. The experimental results indicated the detection limit was 0.3 nmol/L and 1.0 nmol/L in the human plasma and urine samples, respectively.

### 2.4. Electrochemical Method

**Figure 6:**
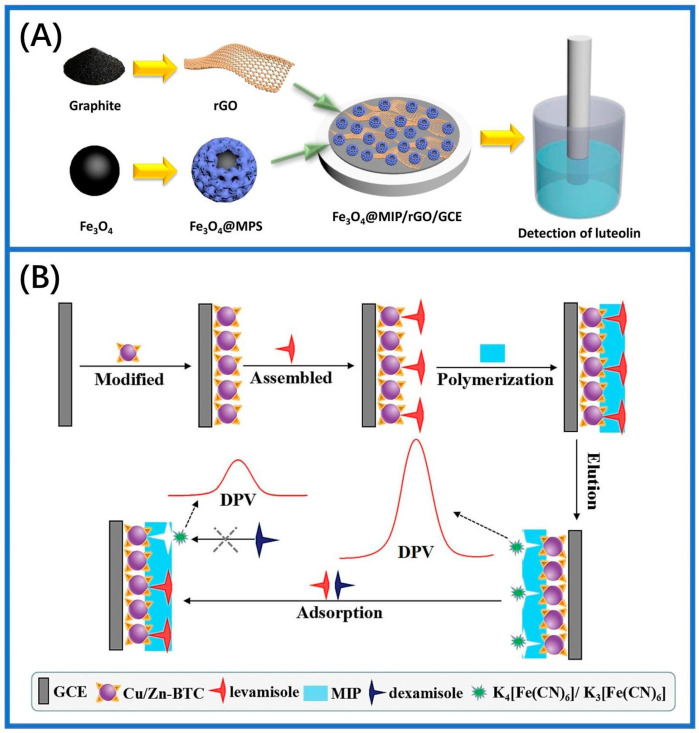
The electrochemical polymerization method for the synthesis of MIMs. (**A**) The fabrication process of Fe_3_O_4_@MIP/rGO/GCE by electropolymerization [84]. (**B**) Preparation mechanism of electropolymerized chiral molecularly imprinted polymer sensor and schematic diagram of levamisole detection [85].

Electrochemical polymerization is a widely used technique for fabricating electrochemical membrane sensors. This method generally involves the creation of MIMs on the electrode surface through electrochemical polymerization or deposition techniques [26,67,68]. The process includes selecting and cleaning the electrode, preparing a casting solution, performing electrochemical polymerization, and subsequently removing the template molecules to yield the MIMs. Electrochemical polymerization offers several advantages over alternative polymerization techniques, such as a faster preparation rate and indirect control over film thickness by adjusting the polymerization solution concentration and scanning cycles. In 2003, Blanco-Lopez and colleagues developed a voltametric sensor to detect vanillylmandelic acid (VMA) with a monomer mixture on a glassy carbon electrode (GCE). This involved spin-coating a layer of MIPs with a blend of monomers (template, cross-linker, solvent, and methacrylic acid), followed by in situ photopolymerization [83]. The membranes obtained exhibited a proper pore size and suitable density, facilitating the diffusion of the analyte toward the electrode surface for effective molecule detection. However, challenges persisted regarding membrane stability and the control of membrane thickness. Furthermore, the selectivity of imprinted membranes in water systems is relatively limited, thereby constraining their potential applications to some extent. Building upon these findings, an electrochemical surface molecular self-assembly strategy was proposed. Figure 6A demonstrates the fabrication process of a glassy carbon electrode coated with a magnetically attracted MIP (Fe_3_O_4_@MIP) to electrochemically detect luteolin. The sensor exhibits a stable performance for a duration of 6 days, with a linear detection range spanning from 2.5 pM to 0.1 μM [84]. As depicted in Figure 6B, a composite chiral sensor was developed by immobilizing MIPs onto the glass carbon electrode surface. The chiral sensor possessed a linear identification range at 5–6000 × 10^−11^ mol/L. In addition, this particular sensor exhibited detection recoveries ranging from 93.8% to 109.0% when used for identifying levamisole in chicken and various authentic samples [85]. Furthermore, enzymatic biosensors with a single-layered membrane structure were developed for the detection of pesticides, utilizing an asymmetric base film made of bromomethylated poly(2,6-dimethyl-1,4-phenylene oxide), BPPO [86]. The biosensor membranes were fabricated utilizing a graphite carbon electrode, which identifies pesticides through the reduction current generated by the enzymatic reaction and subsequent formation of quinone as its end product. Catechol diffused across the solution and permeated into the enzyme domain, where it underwent conversion into quinone and a subsequent reduction atop the electrode’s outer layer. However, an inhibitor-like pesticide resulted in a delayed enzymatic reaction, causing a reduction in the observed current. Meanwhile, another study employed an in situ electrochemical reduction and polymerization process to gradually form MIMs directly on a GCE surface [70]. The prepared electrochemical sensor showed outstanding achievement in determining dihydromyricetin with an extremely sensitive detection threshold of 1.2 × 10^−8^ M.

## 3. Important Applications of MIMs in Various Fields

### 3.1. Electrochemical Biochemical Sensors

The integration of MIT into electrochemical methods facilitates the conversion of reactions occurring on imprinted membranes into monitorable electrical signals, thereby building an efficient and reliable analysis platform [26,87]. MIM functionalized electrochemical sensors are assessed by monitoring changes in current and voltage before and after the analyte enters the imprinted cavity [88,89]. Typically, electrochemical sensors consist of electrodes and electrolytes. The electrodes, a three-electrode system, comprise a reference electrode (RE), a working electrode (WE), and a counter electrode (CE) [90]. Electrolytes are substances that conduct electricity by dissociating into cations and anions when dissolved in water or in their molten state, examples of which include NaCl and PBS. A key feature of electrochemical reactions is the oxidation or reduction of analytes through electron transfer at the electrode. Analytes can be detected either qualitatively or quantitatively by measuring electrical signals such as current, voltage, and resistance. Based on these signals, electrochemical sensors are classified into various types, including amperometric, potentiometric, and impedimetric sensors [91,92,93].

**Figure 7:**
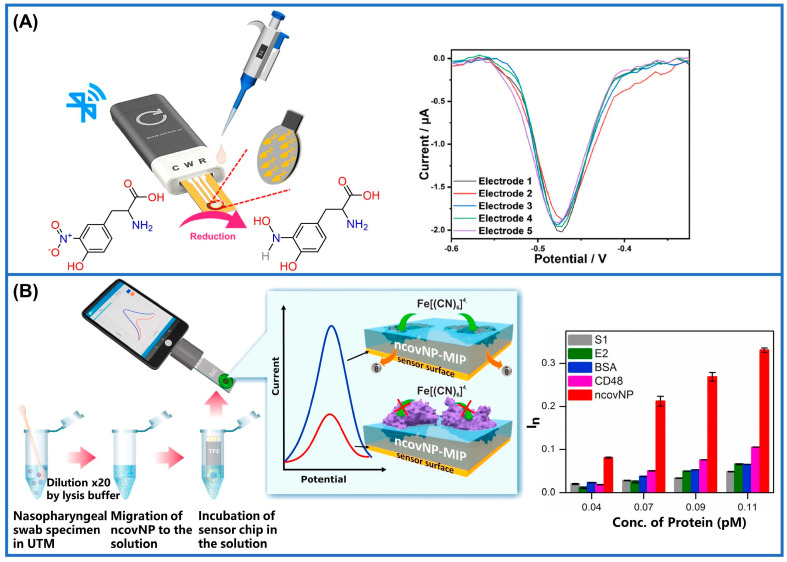
Practical implementations within electrochemical MIM systems. (**A**) Schematic illustration of the detecting process via microsensor and the DPV curves of 3−NT [96]. (**B**) The procedure for electrochemical detection of ncovNP from nasopharyngeal swab samples and the sensor’s selective response towards diverse proteins [104].

Different detection techniques can be utilized during the electrochemical measurement process, including chronoamperometry (CA), differential pulse voltammetry (DPV), cyclic voltammetry (CV), and square wave voltammetry (SWV) [94,95]. As an exemplification of a prototypical point-of-care testing (PoCT) scenario, an MIM-electrochemical method was constructed for the immediate detection of 3-NT by modifying zeolite imidazolate backbone-67 (ZIF-67) on a laser-induced graphene (LIG) substrate (Figure 7A). The sensor is controlled using a tablet or smartphone and requires only 50 μL of a sample solution to complete the test. Figure 7A depicts the DPV results, which showed that the five sensors prepared under identical conditions exhibit excellent repeatability. With a turnaround time of 10 min and minimum detectable level of 6.71 nM, this platform demonstrated promising potential as a point-of-care (PoC) diagnostic tool. By optimizing performance, cost, and biocompatibility, this sensor effectively meets the requirements for routine disease detection [96]. In addition to graphene, GCE is also commonly employed as an electrochemical substrate. An MIM immunosensor was developed for the analysis of AFP by modifying the GCE surface with polythionine (PTh) and gold nanoparticles (AuNPs). The DPV analysis demonstrated that the maximum sensor current remained unaltered in the presence of interferences, indicating its exceptional specificity. Even after storage at low temperatures for seven days, the sensor exhibited a negligible decline in its peak current, showcasing remarkable stability [97]. The identification of catechol by MIT was successfully achieved through the modification of a GCE with reduced graphene oxide. During the imprinting process, catechol was adsorbed onto the surface of polypyrrole (Ppyr), and subsequently, a pollution-free extraction was performed from this mass-produced template. In voltammetry measurements, the sensing device exhibited a broad range of linearity with an LoD at 4.18 nM, demonstrating exceptional levels of selectivity, stability, and repeatability in real sample detection [98]. Capacitance-based molecularly imprinted sensors, also known as impedance sensors, detect changes in capacitance resulting from binding within the analyte-imprinted cavity. In this type of sensor, electrochemical impedance spectroscopy (EIS) is typically employed to investigate the electrochemical process at the electrode/electrolyte interface [99]. For example, researchers have developed MIM electrochemical sensors for the dual detection of sumatriptan (SUM) and paroxetine (PRX) using EIS. They modified pencil graphite electrodes by phosphotungstic acid (PWA) and reduced graphene oxide (rGO) composites, which effectively reduced resistance and enhanced the rate of electron transfer. The EIS results demonstrated that the improved electrode exhibited a high selectivity with imprinting factors of 3.15 for SUM and 3.3 for PRX, respectively. However, the sensor’s measurement relies on static electrochemical technology, requiring sample loading onto the electrode for detection. Consequently, real-time monitoring is unattainable, and offline analysis becomes imperative [100].

MIM electrochemical sensors offer versatile applications beyond pharmaceuticals, including the detection of biological macromolecules like biomarkers related to SARS-CoV-2. For instance, epitope-imprinted polymers synthesized through electropolymerization demonstrated cost-effectiveness and reduced non-specific binding, allowing for the precise recognition of SARS-CoV-2 proteins with a high affinity [101]. By using heptapeptides derived from the SARS-CoV-2 protein with a spike structure as templates for epitope-imprinted polymers, the accurate detection of the parent proteins has been achieved at low picomolar concentrations [102]. Additionally, molecularly imprinted polypyrrole electrodes have proven effective in detecting SARS-CoV-2 spike glycoproteins, showing enhanced levels of affinity and sensitivity compared to non-imprinted counterparts [103]. The development of portable sensors for SARS-CoV-2 antigen detection has emerged, with disposable sensor chips integrating thin-film electrodes and MIMs, demonstrating the real-time analysis of nasopharyngeal swab samples (Figure 7B). These sensors demonstrated exceptional selectivity for ncovNP, achieving a remarkable sensitivity down to 15 fM. This highlights their precision and rapidity in detecting SARS-CoV-2. The sensors’ excellent ability to discriminate interfering proteins, coupled with their rapid response time and user-friendly operation, has greatly enhanced the clinical diagnosis of COVID-19 [104].

As widely known, the sensitivity of MIM electrochemical sensors heavily relies on the quantity of imprinted cavities embedded in the imprinted films [105]. Therefore, the support materials with a large specific surface could expand the imprinted films and generate a greater level of imprinting cavities. Black phosphorus (BP) is a semiconductor material known for its high carrier mobility, significant reducibility, and exceptional electron donor ability, facilitating the facile reduction of chloroauric acid (HAuCl_4_) to AuNPs [106,107,108]. Using these capabilities, AuNP/BP nanosheet-modified GCE (BBNS-AUNP/GCE) sensors were developed for the specific measurement of norfloxacin (NOR). The electrochemical sensor employed linear sweep voltammetry (LSV) for the quantitative analysis of NOR, exhibiting a wide range of responses that are linear in nature (0.1 nM–10 μM) and a low level of identification restriction (0.012 nM). While the sensor exhibits a high level of selectivity, there remains potential for enhancing sensitivity in clinical applications [109]. To address this issue, metal–organic frameworks (MOFs) are employed, which are crystalline support materials with a high porosity, stable backbone, and large surface area [110]. Combining MIMs with MOFs enhances binding capacity and accelerates recombination kinetics. Researchers optimized the surface of GCE, utilizing a copper MOF modified with gold nanoparticles (AuNPs) and nitrogen-doped quantum dots derived from graphene (N-GQDs) (Au@Cu-MOF). The detection limit of this MIM electrochemical sensor platform was as low as 0.0007 ng/mL for patulin. Despite this progress, the sensitivity of MIMs remains constrained by binding site limitations and slow binding/dissociation rates [111]. Typically, the adjustment of structural stability in MOFs often poses challenges, thereby limiting the applicability of certain chemical modifications. Thus, researchers have also used the covalent organic framework (COF) to addresses this issue by incorporating covalently bonded organic units, allowing for the optimization of performance through the selection of diverse organic units and the regulation of synthesis conditions [112]. Conductive MIMs have shown promise in enhancing electrochemical sensor performance. Recently, MIP composites including polyaniline (PANI) were employed in field sensors for an organophosphate pesticide analysis of vegetables, achieving a low LoD of 11.3 nM for the real-time detection of parathion [113]

MIM electrochemical sensors are extensively utilized; however, certain limitations persist. For instance, the utilization of electrochemical probes results in a diminished adsorption capacity, restricting the sensor’s ability to detect molecules beyond the surface’s vicinity. Furthermore, issues such as unstable electrical signals, low conductivity, non-specific adsorption, and other factors necessitate future enhancements in MIM electrochemical sensing technology [92,114].

### 3.2. Surface-Enhanced Raman Scattering

SERS utilizes metal nanostructures to amplify weak Raman signals, enabling the detection of low concentrations or even single molecules [115,116,117,118,119]. SERS offers advantages such as a high sensitivity, spectral resolution, short response time, and molecular fingerprint information. However, interference from substances with similar chemical compositions or bonds may mask target signals, leading to false negatives or detection failure [120,121]. The combination of selective imprinting cavities from MIMs with the SERS method can effectively improve the anti-interference ability of SERS [122]. Furthermore, MIMs can serve as the adsorbent material to bring target molecules closer to the SERS substrate, thereby enhancing the sensitivity of SERS [123,124]. Consequently, the integration of MIMs with SERS technology has attracted significant interest in the fields of biomedicine, environmental monitoring, and food supervision [125].

**Figure 8:**
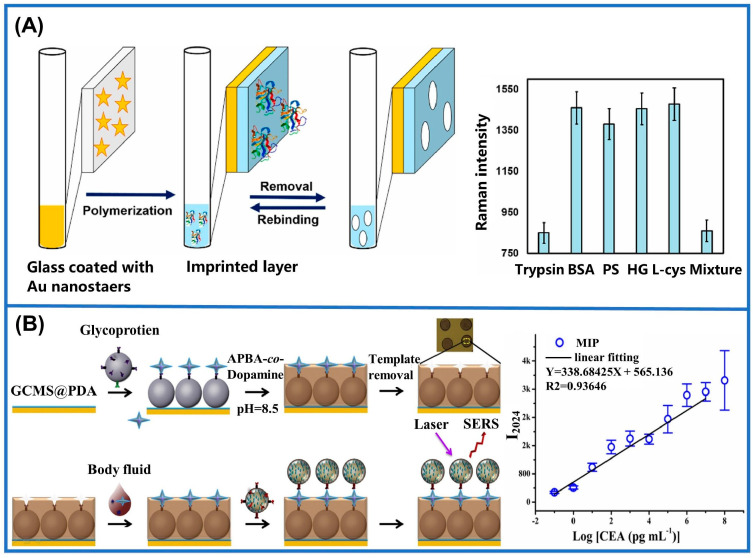
Application of SERS with MIMs in real-world scenarios. (**A**) The schematic depicts the Raman scattering mechanism in a multifunctional capillary SERS sensor and illustrates the sensor’s response to various analytes [129]. (**B**) Construction of the boron−affinity molecularly imprinted sensor and the logarithmic concentration curve of CEA detection based on the average intensity observed at 2024 cm^−1^ [133].

MIM-SERS sensors are generally categorized into “one-step” and “two-step” methods based on their preparation process. In the one-step method, MIMs are directly attached to the SERS substrate, which enriches the target analyte and enhances SERS signals. This approach offers benefits such as simplicity, rapid detection, and minimal solvent consumption. Additionally, one-step MIM-SERS sensors can be further classified into planar and sandwich types. Planar sensors involve directly coating MIMs onto flat SERS substrates, which increases the surface area for a better interaction between target molecules and the substrate, thus enhancing scattering [126,127,128]. For example, multifunctional capillary SERS sensors were developed for protein detection with polydopamine imprinted layers (Figure 8A). The imprinted cavities can effectively capture target proteins while also serving as exclusive pathways for Raman indicators to access SERS substrates. When the imprinting cavities are blocked by target proteins, the reduction in SERS signals can reflect the quantity of captured molecules. Using bovine serum albumin (BSA), pepsin, and hemoglobin as target proteins, the sensor achieved versatility across different analytes [129]. Meanwhile, the detection of BSA can also be achieved with silver nanoparticle (AgNP)-modified graphene sensors. The incorporation of MIMs not only protected the AgNPs, but also enabled their specific binding with BSA. In comparison to conventional approaches, the sensor demonstrated a remarkable improvement in detection sensitivity by seven orders of magnitude. This method, however, still presents opportunities for enhancement, necessitating a further refinement of its materials and preparation techniques to improve its stability and longevity [130]. In addition to large proteins, MIM-SERS sensors are utilized in the identification of minute molecules as well. Through the polymerization of patulin (PAT) triggered by horseradish peroxidase, an Au-polydimethylsiloxane (PDMS)–concrete anodized aluminum oxide (AAO) substrate was constructed. This new approach exhibited an excellent selectivity towards PAT with a lower limit of detection at 8.5 × 10^−11^ M [45]. As for sandwich-type MIM-SERS sensors, they utilize MIMs as the recognition layer to combine them with the target molecules and then generate SERS signals by binding to the upper Raman probes. Owing to the exceptional stability and sensitivity conferred by this sandwich structure, these sensors are highly suitable for detecting biological macromolecules [127]. In 2014, researchers pioneered the creation of a boron-affinity sandwich assay (BASA) for detecting AFP in serum. The target glycoproteins were first extracted by a boron-affinity MIM and then bound to boric-acid-modified SERS labels [131]. In addition, a novel dual-MIP-based immunoassay has been developed for the detection of proteins in complex biological samples. A carbon end epitope-imprinted layer is fabricated atop the assembled underlying material, functioning as a specialized SERS substrate specifically tailored for the intended protein. N-terminal epitope-imprinted Raman response Ag@SiO_2_ nanoparticles are employed as SERS labels in a sandwich structure, resulting in a significant enhancement in and the high specificity of the SERS signal [125]. Utilizing nucleoside diphosphate kinase A (NDKA) antibody as an SERS marker, an MIM-SERS sensor modified with 4-mercaptobenzoic acid (4-MBA) was prepared. The sensor employed polydopamine (PDA) as the substrate of the MIM to capture NDKA, achieving an exemplary test constraint of 0.25 pg/mL in human serum. But the intricate preparation process and the potential interference from substrate fluorescence or scattering present formidable challenges in regards to the sensor’s performance [132]. Except such enhanced Raman tags, the performance of MIM-SERS sensors can also be improved by using tags with specific sites to avoid interference. By employing probes with Raman signals located in the silent region (1800~2800 cm^−1^), researchers avoided optical noise from endogenous compounds within an area with a unique pattern (300~1800 cm^−1^). Due to the excellent anti-interference capability of the sensor, this method can be applied for the PoCT of CEA in real blood (Figure 8B) [133].

In contrast to the complexity of spectra obtained through the “one-step” method, the two-step SERS detection method was developed to separate and enrich the target using MIMs before the SERS detection of the eluted solution. This process effectively removes impurities and interferences, significantly improving the accuracy of results. In MIM-SERS sensors, MIMs function as molecular recognition cavities that fill a solid-phase extraction (SPE) column, specifically enriching the organic compounds from complex matrices [134]. For example, a sensor designed to detect the SERS signals of caffeine using theophylline as a virtual template was developed. The sensor, consisting of AgNPs@ molecularly imprinted SPE (MISPE), a syringe, and a removable microporous membrane, eliminated separate elution steps, thus reducing the overall analysis time. However, the preparation method of the sensor is intricate, necessitating AgNPs’ incorporation into the MIM through precipitation polymerization, thereby potentially compromising the stability and reproducibility of the fabrication process [135]. So MIM-SERS sensors also employ flexible polymer films as substrates, which are characterized by their transparency, flexibility, stability, and low cost. Conventionally, MIMs are coated or poured onto the surface of SERS substrates for detection. Polyvinyl chloride (PVC), as a type of polymer material, has wide applications in various aspects of daily life. As a brilliant example, an AgNP/PVC MIM-SERS sensor was fabricated with PVC as a carrier through the solvent casting technique. The sensor demonstrated undifferentiated detection on both sides, showcasing a robust linear correlation between the SERS intensity and solution concentration at 1595 cm^−1^ [43]. There are also other studies based on MIM-SERS PoCT sensors, such as those utilizing polyvinylidene fluoride (PVDF) for L-tyrosine analysis and an electrospun filament protein membrane (ESM) as a biocompatible scaffold for spiramycin detection [136,137]. Notably, these approaches have all exhibited exceptional nanomolar-level detection limits. However, MIM-SERS sensors still face challenges such as their limited anti-interference capability and complex regeneration processes. To address these issues, researchers are actively developing novel molecular imprinted materials with enhanced levels of specificity, sensitivity, and regeneration performance through precise design and preparation. Furthermore, strategies including portable Raman detectors for PoCT, nanotechnology for efficient SERS substrate fabrication, the integration of intelligent algorithms for data processing, and the optimization of template and monomer identification via boric acid affinity have been proposed. These approaches highlight significant advancements in overcoming the limitations associated with MIM-SERS sensors while offering new avenues to enhance their performance and broaden their application scope.

### 3.3. Surface Plasmon Resonance

Having explored the potential applications of SERS, we now turn our focus on another promising spectral analysis technique: SPR. In SPR-MIM sensors, MIMs selectively capture target analytes, thereby increasing their local concentration on the SPR sensor surface. This leads to a higher signal-to-noise ratio and improves their sensitivity in detection. SPR, originating in the 1990s, is widely used in biomedical fields for detecting ligand–analyte interactions on biosensing chips, facilitating real-time biomolecule analyses [26,138,139]. The principle of SPR involves collective electron oscillations on a metal surface when illuminated by light, generating plasma waves that interact with incident light to enable detection [140]. Its sensitivity to variations within the refractive index of the metal outer layer allows for the precise monitoring of biomolecular interactions via dynamic resonance angle adjustments [141,142]. With the application and development of SPR technology, it has been combined with various techniques to enhance its detection performance [143]. Among these techniques, the combination of MIT with SPR not only confers specificity to the SPR sensing chips but also enhances the sensitivity of MIMs [144,145,146]. This significantly broadens the application areas of SPR and MIT and has garnered significant interest from researchers [147].

**Figure 9:**
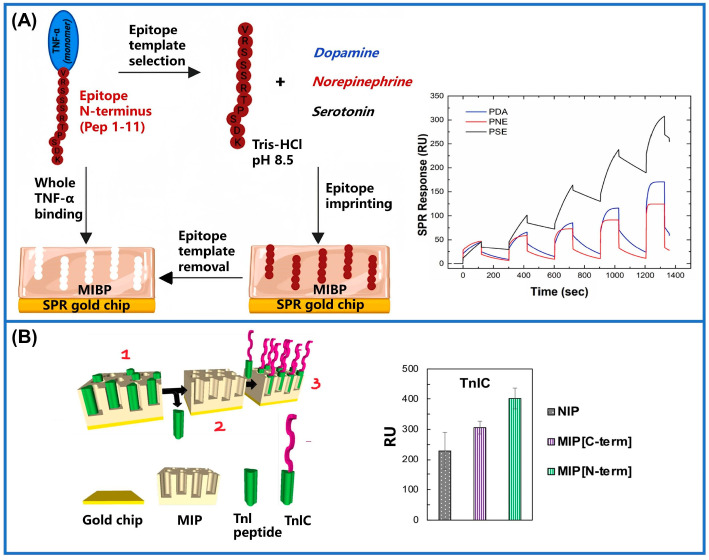
Utilization of SPR in MIMs for real-world applications. (**A**) Fabrication of a printed plasma sensor for detecting tumor necrosis factor-α (TNF-α) and response diagrams for three different analytes [150]. (**B**) Schematic depiction illustrating the procedure of assembling troponin I (TnI) peptide-imprinted sensor and the response of imprinted and non-imprinted sensors at a concentration of 1 mg/L troponin I-C complex (TnIC) [151].

For example, a carbohydrate Antigen-125 (CA-125)-imprinted SPR sensor was developed via electropolymerization utilizing pyrrole (Py) as a monomer. The grafting condition of CA-125 onto the SPR chips can be monitored by changes within the permittivity value of the chip surface and variations in the incident resonance angle of the sensor surface. According to the above principle, the sensor achieves a detection limit for CA-125 of 0.01 U/mL [148]. Researchers also utilized lysine (K) and alanine (A) residues as models to investigate diverse imprinting states. They determined that optimal imprinting occurred when three lysine residues were positioned at the N-terminus. Expanding on these findings, the efficacy of the 3K strategy was further assessed using a peptide template of soluble programmed cell death ligand 1 (PD-L1). Through systematic optimization, the strategy achieved an excellent level of reproducibility (_AV_CV% = 4.5). Ultimately, the SPR sensor constructed based on the N-3K strategy demonstrated an outstanding level of sensitivity (0.31 ± 0.04 ng/mL) in samples of serum from humans, thereby providing a crucial diagnostic tool for healthcare applications [149]. In another study, researchers constructed MIM-SPR sensors employing the naturally occurring neurotransmitter serotonin (SE) as a functional monomer to capture tumor necrosis factor-α (TNF-α) for the first time (Figure 9A). The detection limits of the polyserotonin (PSE) biosensor were 20 times and 13 times higher than those of dopamine (DA)- and norepinephrine (NE)-based MIM sensors, respectively. This indicates that the future use of imprinted PSE as a bionic receptor for PoCT is a promising approach. But from the perspective of clinical and diagnostic applications, further enhancement of its sensitivity is imperative to effectively compete with commercial immunoassay methods and encompass a wide range of clinically relevant concentrations across various conditions and diseases [150]. Similarly, sensors with polynorepinephrine (PNE) as MIMs have also attracted people’s attention. A sensor imprinted two short troponin I (TnI) peptides on PNE, overcoming the disadvantage of whole-protein imprinting in MIMs (Figure 9B). Figure 9B illustrates that the imprinting of N-terminal peptides resulted in a significantly enhanced SPR response, exhibiting an approximate 100 RU increase, when compared to that of TnI-printed C-terminal biosensors. Due to its minimization of non-specific binding, this method provides excellent detection capabilities in the low nanomolar range (7.1 ± 0.6 nM) [151]. MIM-SPR sensors typically exhibit an exceptionally low detection limit and a high sensitivity, rendering them well-suited for the quantification of bacterial factors (such as RoxP) at low concentrations. Compared with non-imprinted SPR chips, the selection of RoxP as the protein imprint resulted in a faster response and higher sensitivity. The sensor exhibited a high affinity constant of 3.3 × 10^−9^ M for the target protein, which can facilitate the early PoC identification and diagnosis of certain oxidative skin diseases. However, the performance of such biosensors can be compromised by signal fluctuations, leading to a substantial standard deviation and diminished robustness [152]. Furthermore, SPR sensors are sensitive to environmental variations, including fluctuations in temperature and humidity, which can potentially impact the sensors’ performance and accuracy. The utilization of MIM-SPR sensors is further hindered by the high costs associated with their preparation and maintenance, limited detection range, as well as their suitability for specific analytes only.

### 3.4. Quartz Crystal Microbalance

A QCM is an extremely precise instrument for measuring mass, capable of monitoring minute changes on the surface of sensing chips [153,154,155,156]. The principle of the QCM is based on the piezoelectric effect, which enables the detection of changes in resonance frequency caused by mass variations on the quartz crystal surface [157,158]. One of the current trends in QCM sensors is their joint application with MIT for sensing [159,160]. Most reported studies attach MIMs to the surface of QCM chips and specifically detect the QCM by introducing specific cavities on the MIMs. When the analyte enters the detection cavities of the MIM, the surface of the chip is compressed and the frequency changes [161,162]. Therefore, the mass signals of the analytes can be converted into frequency signals by the piezoelectric effect of the chips. Recently, numerous studies have focused on the clinical applications of MIM-QCM sensors.

**Figure 10:**
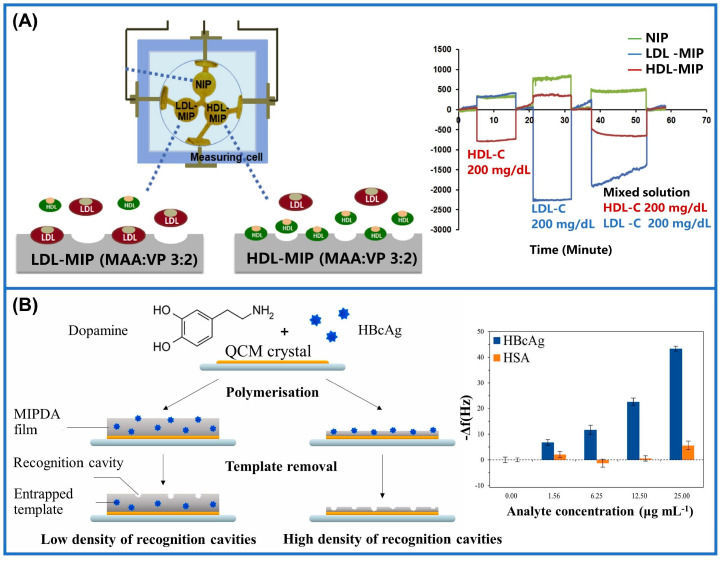
Application of QCM coupled with MIMs for practical use in scientific research. (**A**) Preparation of MIM sensor array for simultaneous assessment of lipoproteins and selectivity curves [163]. (**B**) Schematic illustration of the combination of hepatitis B core antigen (HBcAg) and recognition cavities, along with the corresponding frequency responses of the sensor to HBcAg and human serum albumin (HSA) [165].

An MIM-QCM assay array was constructed for the detection of low-density lipoprotein (LDL) and high-density lipoprotein (HDL) (Figure 10A). Two functional monomers, methacrylic acid and N-vinylpyrrolidone, were chosen to specifically recognize the target proteins, enhancing the sensor’s specificity. The proposed method does not require fasting, thus reducing the pain caused by conventional detection. However, in comparison to commercially available enzyme- and antibody-based methods, the measurement results obtained from this sensor exhibit a higher coefficient of variation, which may impact the accuracy of the findings [163]. A novel enhancement of MIPs, termed electric-field-assisted molecularly imprinted polymers (EFAMIPs), was developed to enhance MIP-modified QCM biosensors. In the existence of a vertical electric field, methacrylic acid is polymerized with immuno-globulin G (IgG) as a template, followed by the template’s removal to obtain EFAMIPs. Studies have demonstrated that EFAMIP-modified QCMs exhibited a significantly higher frequency shift of up to 113.5% in electric field detection when IgG was present, compared to typical MIPs. The ultimate alteration in frequency of EFAMIPs, which determined the IgG detection limit, was enhanced by 12.5% compared to conventional MIPs. Thus, EFAMIPs show promise for selectively detecting IgG in solutions that consist of different categories of immunoglobulins, presenting a novel detection method for clinical applications [164]. In another study, researchers functionalized the surface of QCM crystals with molecularly imprinted polydopamine (MIPDA) membranes for identifying the presence of hepatitis B core antigen (HBcAg) (Figure 10B). The frequency response of the MIPDA-QCM sensor to HBcAg was 7.8 times higher than that of human serum albumin (HSA) under the same conditions. Due to the sensitivity and simplicity of its operation, this sensor has practical application value in the PoCT of hepatitis B. Nevertheless, the stability of the sensor’s biometric elements (MIPDA films) may pose a potential concern as prolonged usage and storage could result in film degradation and failure, thereby impacting the sensor’s performance and reliability [165]. Hence, scholars developed computationally assisted simulation methods, aiming to facilitate the design of MIMs with enhanced levels of stability and sensitivity. As a brilliant example, hexestrol (DES) was detected through an MIM-QCM sensor, for which researchers optimized the ratio of functional monomers and cross-linkers by simulating the molecularly imprinted self-assembly system of the DES. Guided by their calculations, they found that the optimal molar ratio of DES to methacrylic acid was one to five. At this point, the sensor had a strong affinity for DES with a test constraint of 2.63 ng/mL. After numerous cycles, however, the sensor’s detection rate experienced a significant reduction. It is speculated that the internal pore structure of DES-MIMs on the surface underwent some damage during electrode cleaning, thus leading to an escalation in the error margin of the detection outcomes [166]. As another small molecule, L-tryptophan was monitored by in situ polymerization using methyl methacrylate as a monomer. After optimizing the quantity of the agent used for cross-linking, the MIM containing 10 mmol ethylene glycol dimethacrylate (EGDMA) possessed the best adsorption ability for L-tryptophan. The modified sensors exhibited an excellent level of selectivity and low response level to interferents such as D-tryptophan and ascorbic acid [161].

Additionally, MIM-QCM sensors can be utilized for the detection of other analytes, such as N-hexanoyl-L-homocysteine lactone, insulin, oxidized LDL, very-low-density lipoprotein (VLDL), and adenylate kinase 1 [167,168,169,170]. Analytical results from real samples demonstrate the great potential of MIM-QCM sensors in the detection field. However, the QCM is susceptible to environmental vibrations and is prone to surface contamination from grease and dust. Primarily employed for mass measurements in liquids, the QCM has a limited capability in measuring solid samples. Currently, molecular simulation computing, virtual template molecules, and computer-aided methods have been employed to design highly specific MIMs that cover the surface of QCM chips to alleviate the issues arising from the inherent shortcomings in QCM technology. It is expected that the advancement of MIM-QCM sensor technology will progressively overcome these limitations, thereby effectively harnessing the benefits offered by both technologies.

### 3.5. Ion-Sensitive Field-Effect Transistors

In recent years, FET-type biosensors leveraging various nanomaterials—such as silicon nanowires, graphene, and transition metal disulfides—have burgeoned. These biosensors have emerged as potent tools for detecting diverse biomolecular targets, both within clinical laboratory settings and for PoC diagnostics.

FETs are electronic devices designed for signal transmission and amplification, initially conceived in 1947 as a quantitative method for biomolecule detection. FET sensors comprise three distinct electrodes: the source, drain, and gate [171,172,173]. MIMs and FETs can be combined in two ways: firstly, by coating the MIMs on the gate, where the adsorption/removal of the template from the gate has an impact on the overall voltage applied to the gate, thereby influencing transistor conduction. MIMs can be combined with the two-dimensional materials in the transistor channel, allowing for the detection of electrical signal changes before and after the combination of materials, which negates the need for MIM conductive performance. FETs operate by applying a voltage to the gate, which induces electron flow from the source to the drain. Analytes affect charge transport in the channel, making the device responsive to the electric field generated by the gate electrode. This principle allows for the conversion of biological or biochemical signals into electrical signals, which is fundamental for biomolecule detection using transistors [174,175,176]. As sensors, transistors offer a straightforward, cost-effective, portable, and real-time detection mechanism, enabling interaction with a broad range of biomolecules. The sensitivity of these sensors is determined by the transistor’s signal conduction and amplification capabilities. Over time, FET technology has evolved, leading to the inclusion of various structures and signal generation/amplification methods, including ion-sensitive FETs (ISFETs) [177,178], organic electrochemical transistors (OECTs) [179,180,181], organic FETs (OFETs) [182,183,184], and so on. Integrating transistors with sensors and MIMs leverages both the signal transmission and amplification abilities of the transistors and the high selectivity of the MIMs. This combination addresses challenges such as interference from complex bodily fluids by using the specific affinity of target biomolecules and the imprinted cavities of the MIMs. The addition of surface nanostructuring can further enhance the affinity of MIM structures for gate electrodes [185], while the precise adjustment of their surface roughness to match the size of analyte molecules can improve their biosensing performance [186].

**Figure 11:**
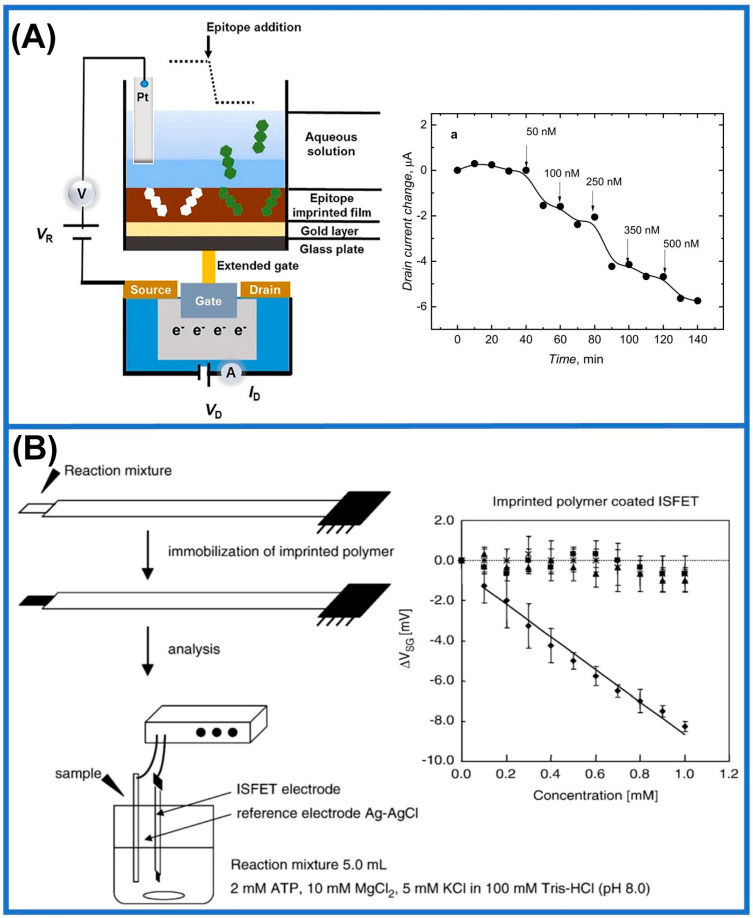
Practical FET applications in the field of MIMs. (**A**) Principle of preparation and detection of MIP-based extended-gate FET (EG−FET) [187]. (**B**) Preparation process of the molecular-imprinted ISFET and generation principle of imprinted cavies on the ISFET gate [188].

The integration of MIMs and FETs presents a powerful approach to chemical sensing and detection. Sensors created through this combination provide several benefits, including an enhanced specificity level, high sensitivity, rapid response times, and label-free detection. Firstly, the high selectivity of MIMs for specific target molecules reduces disturbance caused by additional substances present in the sample matrix, thereby improving the accuracy of FET detection. Secondly, FET sensors’ rapid response capability enables an immediate change in conductivity when target molecules bind to the MIM-functionalized FET surface, allowing for swift detection. Lastly, unlike conventional FET biosensors, MIM-functionalized FET sensors possess the potential for miniaturization and integration into compact, portable devices dedicated to PoC diagnostics, environmental monitoring, and field inspection applications. Recent studies have reported numerous applications of MIMs and FETs. For instance, one study integrated conductive MIMs with extended gate FET (EG-FET) sensors to identify specific antigenic regions of matrix metalloproteinase-1 (MMP-1) protein, a biomarker of idiopathic pulmonary fibrosis (IPF) [187]. This prepared sensor selectively detected MMP-1 protein within the concentration range of 50 to 500 nanomolar in serum (Figure 11A). Meanwhile, researchers developed a novel molecularly imprinted electrochemical sensor based on ISFET devices for urea detection [178]. MIMs were synthesized by the surface photopolymerization of ISFET devices using PMMA and urea as functional polymers and molecular templates, respectively. The LoD of the MIMs-modified ISFET sensor was determined to be 1.0 × 10^−4^ M. Figure 11B displays that a cyclic adenosine monophosphate (cAMP) mimetic ISFET sensor was fabricated using cyclic AMP as the molecularly imprinted material [188]. The ISFET electrode was coated with a polymer film that had been imprinted, leading to the development of sensors capable of effectively binding and selectively detecting cAMP in aqueous solutions. Furthermore, another study used an inorganic sol-gel polymerization mixture of titanium (IV) butanol–carboxylate complexes to create a thin layer on the surface of an ISFET gate [189]. The device provides repeatable measurement results at ambient temperatures with a voltage of ± 2 mV.

**Figure 12:**
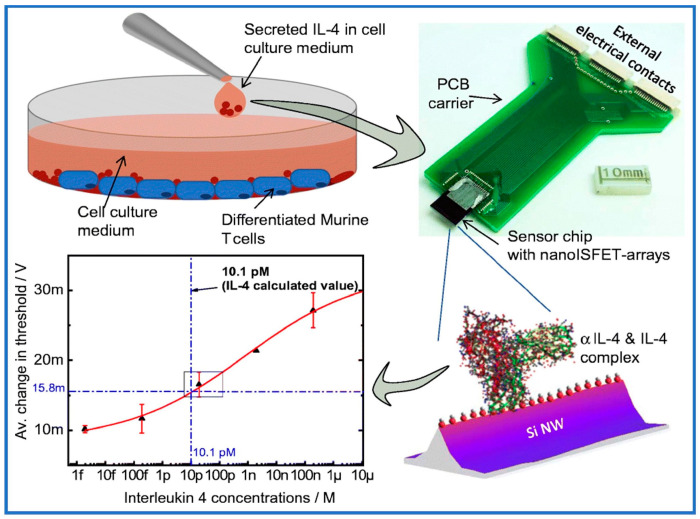
Utilization of nanoISFET sensors in PoC applications. Development of nanoscale ISFET arrays with point-of-care capability for ultra-sensitive detection of cytokines in cell cultures [190].

The proof-of-concept readiness of top-down fabricated nanoscale Si-based ISFET arrays (nanoISFETs) as a biosensor device for the label-free electrical detection of cell-secreted IL-4 and IL-2 was demonstrated (Figure 12) [190]. Increasingly, studies indicate that the combination of MIMs with FETs is not only widely applicable in trace detection but also addresses the stability issues associated with traditional antigen–antibody transistors. However, there is a paucity of literature on the combined application of these two technologies. This may be attributed to the organic reagents employed in the synthesis of MIMs and the cumbersome synthesis steps. Table 2 displays a compilation of diverse applications utilized in MIM biosensors.

## 4. Challenging and Limitations

MIMs present immense potential for enhancing the progression of all-solid-state biochemical sensors by virtue of their selective recognition and stability. Nonetheless, several noteworthy challenges and constraints persist in their practical utilization that necessitate careful attention and resolution. The foremost challenge lies in the imprinting efficiency of MIMs. Factors like the choice of functional monomers, the template-to-monomer ratio, and polymerization conditions profoundly influence the binding uniformity and specificity of MIMs. While significant strides have been made in polymerization techniques and functional monomer design, further enhancements in imprinting efficiency require extensive research endeavors.

Moreover, the long-term resilience of MIMs presents significant hurdles in real-world applications. Polymer degradation under varying environmental conditions such as pH, temperature, and humidity, along with shifts in binding affinity, also directly impact the reliability and longevity of sensors. Furthermore, ensuring reproducibility and standardization in MIM synthesis is paramount in current research endeavors. Consistency in MIM synthesis and sensor performance across different production batches and laboratories is pivotal for driving commercialization initiatives forward.

In summary, addressing these challenges necessitates collaborative efforts and innovative strategies from materials scientists, chemists, and sensor engineers. Overcoming these obstacles successfully will pave the way for broader advancements and applications of MIMs in diverse fields including environmental monitoring, food safety, and medical diagnostics.

## 5. Conclusions and Prospects

Through a comprehensive analysis of current research and methodologies, this paper unveils the unique benefits and potential applications of combining MIMs on 2D solid-supported substrates with state-of-the-art sensing technologies. This integration facilitates interdisciplinary research and has a significant impact on materials science and clinical testing applications. Supported by concrete examples and experimental data, these platforms enable the simultaneous detection of multiple disease markers and their seamless integration with various diagnostic technologies, including electrochemical and optical sensing, as well as mass spectroscopy and semiconducting detection. These cutting-edge sensing technologies facilitate the development of rapid on-site detection for precision diagnosis and personalized medicine.

The preceding discussion underscores the extensive utilization of MIMs across electricity, optics, and mechanics, yet PoC biosensors within the realm of FETs remain notably absent. Notably, sensors built upon FET technology offer compelling attributes such as their compactness, facile scalability, broad applicability, and cost-effectiveness. Therefore, we are confident that this research area will attract the interest of researchers and will be widely used in the future. A recent development in MITs was based on a molecular-imprinted self-assembled monolayer, for which a molecular-thick recognition layer was adsorbed on the nanostructured surface, providing a highly effective PoC diagnostic platform [191]. This technology can eventually be integrated with the processing of 2D solid-supported substrates to provide fast, accurate, and highly specific detection.

MIT’s increasing prominence due to its numerous advantages has been recognized. However, despite years of development, commercializing MIT innovations remains a slow and arduous process. Early proponents of using MIMs in separation science established several companies with some success. Nevertheless, the majority of commercial applications primarily focus on material separation rather than sensor technologies. While MIMs demonstrate a high separation performance, their utilization remains primarily confined to academic settings, with their integration into industrial applications still in its nascent stages. We posit that several factors impede their translation into industrial settings.

Firstly, in the development of MIM-based sensors, the occurrence of false-positive signals resulting from the non-specific binding of polymers to templated materials diminishes the sensors’ accuracy in practical applications. Due to the inherently adsorbent nature of polymers, some degree regarding the engagement with the molecule of interest is inevitable. Moreover, the presence of inhomogeneous binding sites can result in the rebinding of substances, limiting the relevant applications of polymers. Additionally, the employment of numerous environmentally unfriendly solvents and reagents in the manufacturing process poses challenges in adhering to stringent environmental regulations aimed at waste reduction and environmental protection. Clearly, the integrated application of MIMs is imperative to foster their widespread adoption and market penetration in sensing applications. As a result, there is growing interest among scientists and manufacturers in investigating production methods that are more eco-friendly and improving the effectiveness of separation processes to increase efficiency. We anticipate the imminent emergence of commercial applications leveraging MIM-based sensing technology.

Future research should prioritize the development of novel cross-linking agents and functional monomers to enhance the selectivity and specificity of MIMs. Additionally, there is a need to explore environmentally friendly materials and methods for MIMs to minimize environmental impact and promote sustainable sensing technologies. Research efforts should also focus on identifying 2D solid-supported substrates with enhanced stability and durability levels to further elevate sensor performance. The integration of MIMs and 2D solid-supported substrates can enable the creation of multifunctional sensing platforms capable of simultaneously detecting multiple target molecules. This advancement significantly broadens the application scope and practical utility of sensors, catering to diverse detection needs across various fields. In terms of applications, leveraging the advancements in the Internet and smart device technologies, MIMs can be integrated with smart sensing technologies to develop portable, real-time detection sensors. These sensors hold immense potential for widespread use in clinical diagnostics, environmental monitoring, and food safety, delivering timely and precise detection outcomes. Finally, it is investigated how to realize the large-scale preparation and commercial application of composite sensors with molecularly imprinted membranes and 2D solid-supported substrates. Optimizing production processes and reducing costs are crucial steps towards enhancing the competitiveness of these sensors in practical applications, thereby fostering the technology’s adoption and market penetration. By advancing research along the outlined directions, the application potential of MIMs on 2D solid-supported substrates will expand significantly, presenting new opportunities and challenges for the development of all-solid-supported biochemical sensors. Future innovative research holds the promise of accelerating progress in this field, ultimately benefiting various sectors of society.

## Figures and Tables

**Table 1:** Comparison of MIM synthetic methods.

Methods	Merits and Demerits	Synthetic Process	Imprinted Time	Removal Template	References
In situ polymerization	Merits: simple preparation process; low porosity of MIMs; high rigidity of MIMs Demerits: poor membrane permeability; the templates are hard to clean	The membrane that is supported by glass or other substances is immersed in the solution, which is composed of template molecules, functional monomers, and cross-linking agents. Following the polymerization reaction, the template molecules are eluted.	45 min–1 h	The mixture of methanol/acetic acid (90/10) is washed for 4 h	[57,58,59]
Phase-inversion	Merits: improves membrane flux; recognition cavities directly in polymeric materials Demerits: complex synthesis process	A mixture of template molecules and functional monomers is dropped on the carrier and placed in a coagulation bath or an inert gas atmosphere.	Almost 12 h	Methanol/acetic acid mixture (9:1, *v*/*v*) is repeatedly washed until the UV detector cannot detect the template molecule (ARU).	[60,61,62]
Sol-gel	Merits: excellent selectivity; rapid adsorption rate Demerits: longer preparation time; environmentally unfriendly reagents using	Functional monomers and cross-linking agents are initially dissolved in a solvent. Subsequently, a polymer network is formed by polymerization or cross-linking reactions. Finally, the template molecules are removed, resulting in the formation of MIMs.	Almost 24 h	The mixture of methanol:acetic acid (80:20) is washed for 4 h	[63,64,65,66]
Electrochemical	Merits: fast preparation speed; controllable thickness; direct preparation on the electrode surface Demerits: MIMs are brittle; template molecules cannot be aligned	The electrodes are cleaned and immersed in an electrolyte solution containing template molecules, which are used to electrochemically polymerize the imprinted film.	A few minutes	Soak in acetic acid/methanol mixture (*v*/*v* = 1:1) for 10–30 min	[26,67,68,69,70]

**Table 2:** Compilation of diverse applications of MIM biosensors.

Transducer	Nanomaterial	MIM Synthesis Method	Analyte	Detection Range	Detection Limit	References
Electrochemical	ZIF-67/LIG	Electropolymerization	3-nitrotyrosine	0.04 μM–100 μM	6.71 nM	[96]
	PTh/AuNPs/GCE	Electropolymerization	α-fetoprotein	0.001 ng/mL–800 ng/mL	0.8138 pg/mL	[97]
	rGO@Fe_3_O_4_/GCE	Sol-gel	Catechol	1 μM–50 μM	4.18 nM	[98]
	Polyoxometalate/rGO/PGE	Sol-gel	Sumatriptan Paroxetine	0.02 μM–3 μM 0.005 μM–2.2 μM	4.0 nM 0.7 nM	[100]
	Gold wire electrodes	Electropolymerization	SARS-CoV-2	\	\	[101]
	Gold SPRi chips	Electropolymerization	Receptor binding domain of SARS-CoV-2 apical protein	2.5 μM–50 μM	\	[102]
	Pt electrode	Electropolymerization	SARS-CoV-2 spike	0 μg/mL–25 μg/mL	\	[103]
	Disposable sensor chip/TFE	Electropolymerization	SARS-CoV-2 antigen	2.22 fM–111 fM	15 fM	[104]
	BPNS-AuNP/GCE	Electropolymerization	Norfloxacin	0.1 nM–10 μM	0.012 nM	[109]
	Au@Cu-MOF/N-GQDs/GCE	Electropolymerization	Patulin	0.001 ng/mL–70.0 ng/mL	0.0007 ng/mL	[111]
	FUN-PANI/GC	Sol-gel	Parathion	0.034 μM–0.0187 mM	0.0113 μM	[113]
SERS	AuNPs/Glass	In situ polymerization	Protein biomarkers	0.01 μg/L–1000 μg/L	4.1 ng/L	[129]
	G/Ag	Sol-gel	Bovine serum albumin	\	10 pM	[130]
	Au/PDMS/AAO	In situ polymerization	Patulin	0.5 nM–1 μM	0.085 nM	[45]
	AuNPs SAM/Glass	In situ polymerization	Protein biomarkers	0.1 ng/mL–10 μg/mL	\	[125]
	AuNPs/Si	In situ polymerization	Nucleoside diphosphate kinase A	100 pg/mL–10 ng/mL	0.25 pg/mL	[132]
	GCMS	In situ polymerization	Carcinoembryonic antigen	0.1 pg/mL–10 μg /mL	0.064 pg/mL	[133]
	AgNPs@MISPE	Precipitate polymerization	Caffeine	0.1 mmol/L–0.35 mmol/L	100 ng/L	[135]
	AgNPs/PVC	In situ polymerization	Norfloxacin	1 nM–0.01 mM	1 nM	[43]
	Ag/CNTs	Sol-gel	Spiramycin	0.01 nM–1 μM	0.01 nM	[136]
	WA/pDA/PVDF	Precipitate polymerization	L-tyrosine	1 nM–1 mM	1 nM	[137]
SPR	Au-SPE	Electropolymerization	Carbohydrate antigen 125	0.01 U/mL–500 U/mL	0.01 U/mL	[148]
	Au chip	Phase-inversion	Soluble programmed cell death protein 1 ligand	0.25 μg/mL–10 μg/mL	4.2 ± 0.4 ng/mL	[149]
	Au chip	In situ polymerization	Tumor necrosis factor-alpha	\	21 ± 4 pmol/L	[150]
	Au chip	In situ polymerization	Norepinephrine	\	193 ± 30 pM	[151]
	Au chip	In situ polymerization	RoxP	\	0.23 nM	[152]
QCM	Au-QCM electrodes	Sol-gel	LDL-C HDL-C	3 mg/dL–400 mg/dL 8 mg/dL–200 mg/dL	3 mg/dL 8 mg/dL	[163]
	Au-QCM electrodes	Sol-gel	Immunoglobulin G	\	\	[164]
	Au-QCM electrodes	In situ polymerization	Hepatitis B core antigen	0.88 μg/mL–25 μg/mL	0.88 μg/mL	[165]
	QCM electrodes	Sol-gel	Diethylstilbestrol	50 ng/mL–350 ng/mL	2.63 ng/mL	[166]
	Au-QCM electrodes	Sol-gel	L-tryptophan	0.0012 μM–0.204 μM	0.73 ng/mL	[161]
	Au-QCM electrodes	Sol-gel	Very-low-density lipoprotein	2.5 mg/dL–100 mg/dL	1.5 mg/dL	[167]
	Au-QCM electrodes	Sol-gel	Oxidized low-density lipoprotein	86 μg/dL–5600 μg/dL	86 μg/dL	[168]
	Au-QCM electrodes	Sol-gel	Insulin	0.008 ng/mL–1.0 ng/mL	1.58 pg/mL	[169]
	QCM electrodes	In situ polymerization	N-hexanoyl-L-homocysteine lactone	1 μM–50 μM	1 μM	[170]
FET	Au/Glass	Electropolymerization	Matrix metalloproteinase-1	50 nM–500 nM	20 nM	[187]
	ISFET electrode	Sol-gel	Urea	0.1 mM–0.1 M	0.1 mM	[178]
	ISFET electrode	Sol-gel	cAMP	0.1 mM–1.0 mM	0.1 mM	[188]
	ISFET electrode	Sol-gel	Chloroaromatic acids	0.08 mM–1 mM	0.15 mM	[189]

## Data Availability

No new data were created or analyzed in this study. Data sharing is not applicable to this article.
